# High yield of retrospective active case finding for leprosy in Comoros

**DOI:** 10.1371/journal.pntd.0010158

**Published:** 2022-03-03

**Authors:** Nimer Ortuño-Gutiérrez, Aboubacar Mzembaba, Abdallah Baco, Sofie M. Braet, Assoumani Younoussa, Zahara Salim, Mohamed Amidy, Saverio Grillone, Aouladi Said, Bouke C. de Jong, Jan Hendrik Richardus, Epco Hasker

**Affiliations:** 1 Damien Foundation, Brussels, Belgium; 2 National Tuberculosis and Leprosy Control Program, Moroni, The Union of Comoros; 3 Institute of Tropical Medicine, Antwerp, Belgium; 4 University of Antwerp, Antwerp, Belgium; 5 Department of Public Health, Erasmus MC, University Medical Center Rotterdam, Rotterdam, The Netherlands; Hospital Infantil de Mexico Federico Gomez, MEXICO

## Introduction

Cavaliero and colleagues [1,2] describe a high yield of retrospective active case finding for leprosy in Cambodia, which has a fairly low level of endemicity. We conducted a comparable survey in high prevalence villages on the island of Anjouan, Comoros, which is highly endemic for leprosy with an annual incidence rate of 550 per 1,000,000 population reported for 2019 [3]. Over the last 15 years, leprosy incidence on Anjouan has been consistently high, approximately 912 per 1,000,000 per year on average. For years, the National Leprosy Programme (NLP) has been conducting so-called “mini campaigns” or “skin camps,” based on the “camp approach” in which inhabitants of villages are invited for screening for all kinds of skin conditions in a central location [4]. Yet, when door-to-door screening was conducted in some of these villages served earlier with mini campaigns, very high numbers of new leprosy cases were detected [5]. On Anjouan, in addition to mini campaigns, household contacts of leprosy patients diagnosed are either visited in their homes by nurses trained in leprosy or invited to present to Primary Health Care (PHC) facilities for leprosy screening. In the ongoing PEP4LEP trial, conducted elsewhere, the effectiveness of the skin camp approach is being compared to self-presentation of contacts at health centers [6].

### The intervention

Whereas screening of household contacts is typically conducted soon after a leprosy patient has been newly diagnosed, we opted for screening household contacts of all patients diagnosed in the preceding 4-year period, i.e., since January 1, 2017. The villages selected were not included in the large ongoing PEOPLE trial on postexposure prophylaxis for leprosy, covering 32 of the most endemic villages on Anjouan, either because of their large population sizes or because at village level leprosy incidence was below average.[7] They were visited in December 2020. Under the coordination of the NLP, a sensitization session of community leaders was organized to enhance the acceptability of the screening. Prior to the visits, line listings were prepared by village of all leprosy patients registered since January 1, 2017. For each household to be visited, a form was created specifying the village, name, age, and gender of the index case and 15 lines to record contacts, 1 line per contact with a preprinted unique barcode. A customized app in Open Data Kit (ODK) was used for data collection. Besides sociodemographic and clinical data, the app allows recording the geographic coordinates of the household and scanning the barcodes and entering screening results for each individual. In total, 226 index case households were included, but additional forms were provided to screen and record results from surrounding households as deemed necessary by the field team and/or requested by the village members.

### Ethics statement

The NLP authorized the use of anonymized aggregated secondary data for retrospective analysis and publication. Additional approval for publication was obtained from the Institutional Review Board (IRB) of the Institute of Tropical Medicine (ITM), Antwerp (Approval number: 1541/21). Oral consent of participants was obtained as per guidelines that includes active case finding as routine programmatic activity. To avoid identification of affected households, a random error of 25 meters was added to the map shown in Fig 2.

## Results

**Fig 1:**
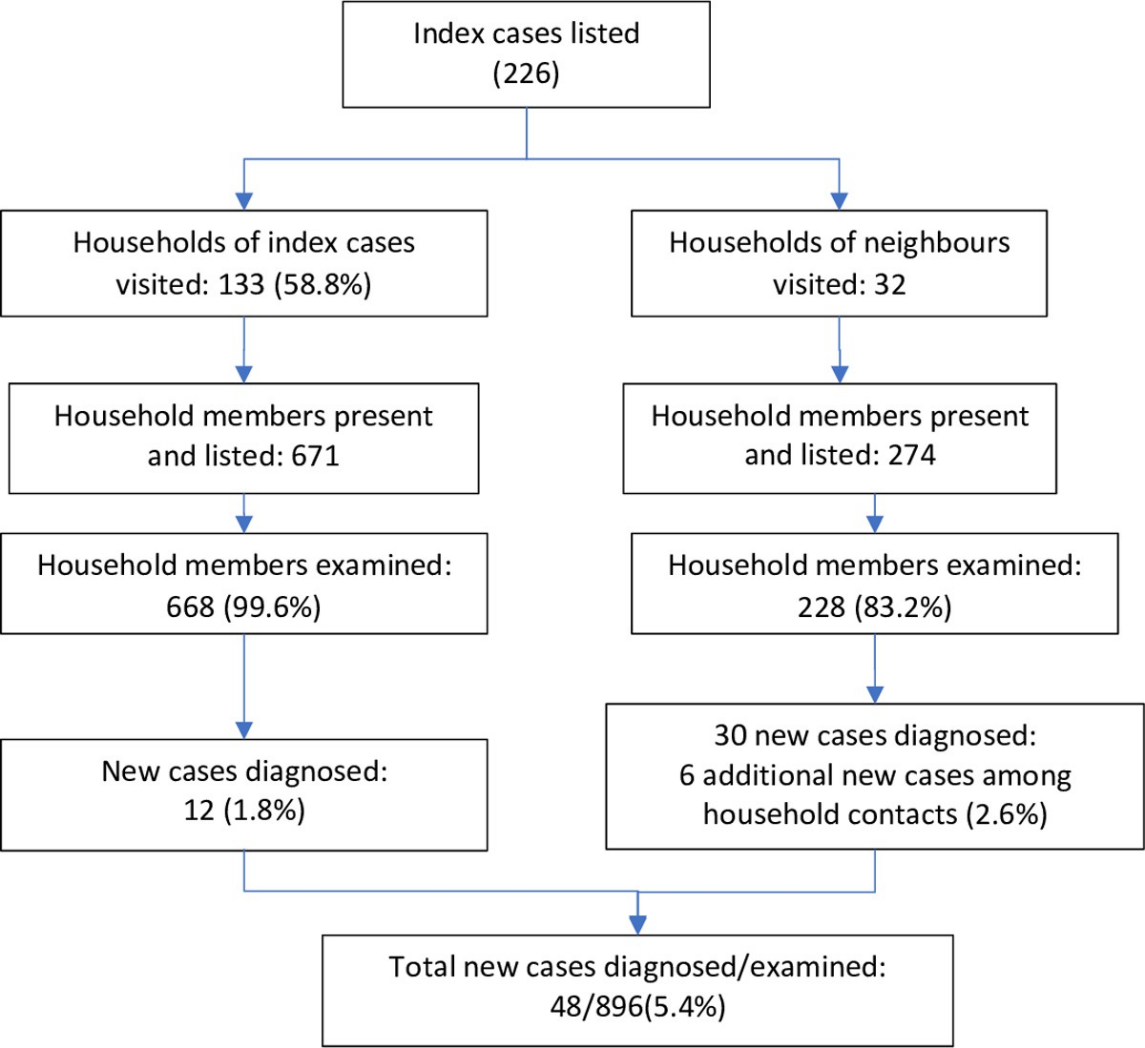
Flowchart of households screened for leprosy, Anjouan, December 2020.

Between December 1 and December 17, 2020, 133 out of 226 index case households listed (58.8%) were visited, as well as 32 other nearby households. Households not visited were either absent or did not agree to screening. Most households (66.1%) were visited during the final 4 days of the survey. Over this period 945 persons were recorded: 671 household contacts and 274 neighborhood contacts. Out of those, 668 were screened, including 471 household contacts of index cases, among whom 12 new leprosy patients were diagnosed. A total of 32 neighborhood contacts screened were among those who spontaneously presented to the team because of skin lesions that were suspected to be leprosy. They were visited in their homes for further examination along with their household contacts. Thus, 30 new leprosy cases were identified among the self-presenting neighborhood contacts as well as 6 additional new cases among their household contacts (Fig 1).

**Fig 2:**
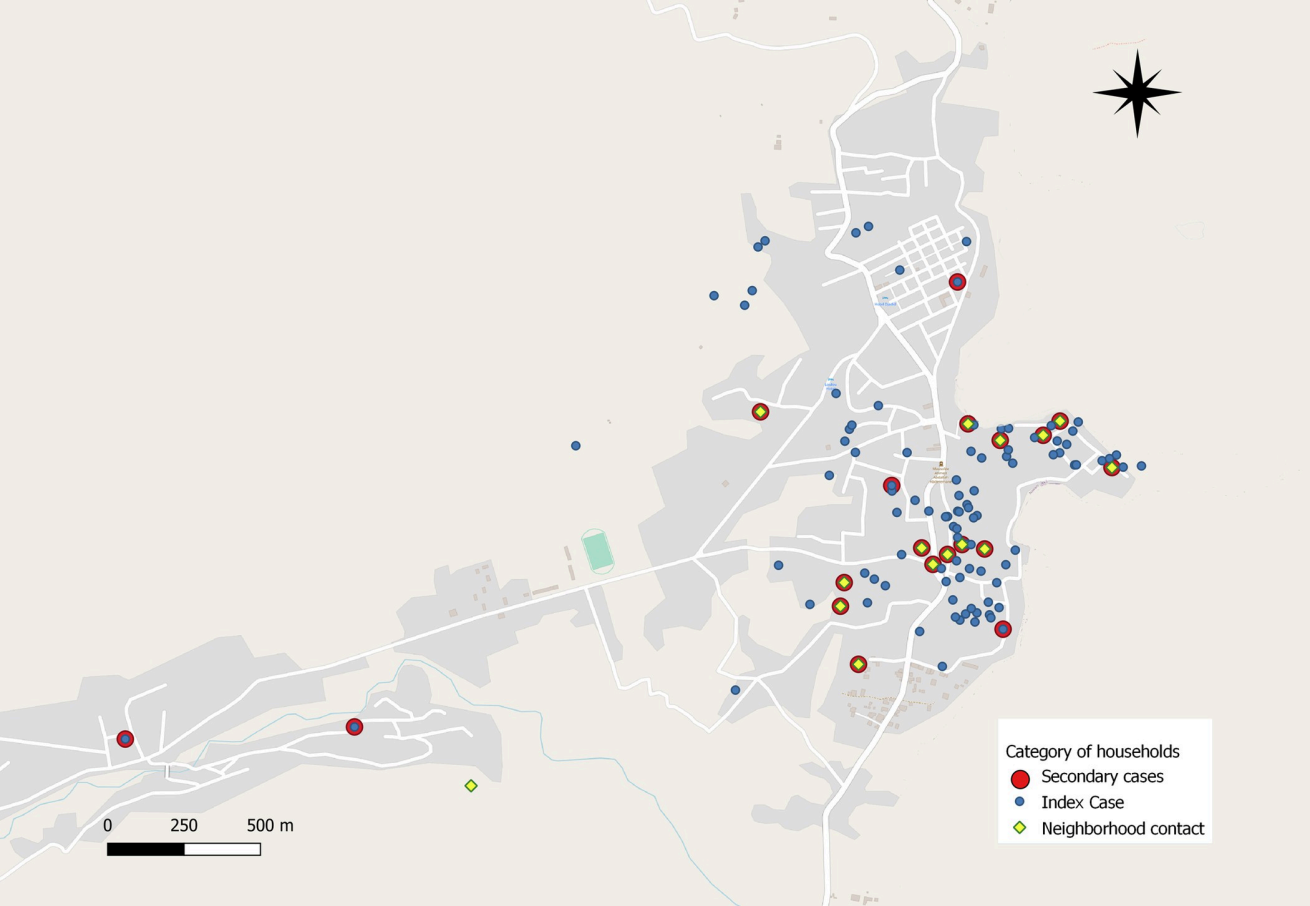
Map of a village with the households screened for leprosy (a random error of 25 meters was added to protect privacy of households screened). We used OpenStreetMap as a base layer for this map (https://www.openstreetmap.org/#map=8/50.510/4.475).

Altogether, 48 new leprosy patients were detected, all autochthonous. A total of 12 were detected among household contacts and the remaining 36 among neighborhood contacts. Of the 48 cases identified, 13 (27.1%) were multibacillary (MB) and 35(72.9%) were paucibacillary (PB). Median age was 18 years (IQR 8 to 34). A total of 19 (40%) were below 15 years of age, of those 7 were in the age category of 4 to 9 years, and the remainder were 10 to 15 years old. None of the patients presented with grade 1 disability, but 2 (4.2%) had grade 2 disabilities. The median distance to index cases among nonhousehold contacts was 98 meters (IQR 60 to 217 meters). Fig 2 below shows the distribution of households visited and cases identified for the largest village included in the survey.

Among the 133 index case households, 61 were households of PB cases and 72 belonged to MB cases. Out of 681 contacts recorded in those households, 252 out of 358 (70.4%) from PB households and 228 out of 323 (70.6%) from MB households were screened. In total, 11 out of 12 cases among household contacts were from MB households, and 1 was from a PB household (OR 12.6, 95% CI 1.6 to 99.6).

## Discussion

This screening effort proved highly effective. Overall, the leprosy new case detection rate was 18.0 per 1,000 (95% CI 9.3 to 31.1) among 668 household contacts examined, most of whom had already been visited previously, but longer ago. An additional 30 cases were found among persons presenting spontaneously, and upon screening 228 of their household contacts, 6 more cases were found, equivalent to a residual new case detection rate of 26.3 per 1,000 (95% CI 9.7 to 56.4). Being a contact of an MB index case was strongly associated with the probability of developing leprosy; this is similar to the findings in Bangladesh where household contacts of highly skin smear-positive index cases (MB patients) had more than 3 times higher risk of developing leprosy. [8]

This experience reinforces the notion that contact screening should not be a one-time effort but is worth repeating after some years, as was highlighted by Cavaliero and colleagues in a low endemic context. In the largest village shown in Fig 2, leprosy cases are clearly clustered in the southeastern part of the village. In such high prevalence zones, even repeated (e.g., annual or biannual) door-to-door screening could be considered until clear signs of decline are demonstrated (e.g., until no more children are found among newly diagnosed leprosy cases).

Since household contacts clearly are at extremely high risk, postexposure prophylaxis is strongly recommended [9]. In highly affected neighborhoods, even blanket coverage with postexposure prophylaxis should be considered [10]. The tools used allow to easily identify such high-risk areas and can guide further screening efforts, even under programmatic conditions.
